# A novel sheet-like virus particle array is a hallmark of Zika virus infection

**DOI:** 10.1038/s41426-018-0071-8

**Published:** 2018-04-25

**Authors:** Jun Liu, Brandon A. Kline, Tara A. Kenny, Darci R. Smith, Veronica Soloveva, Brett Beitzel, Song Pang, Stephen Lockett, Harald F. Hess, Gustavo Palacios, Jens H. Kuhn, Mei G. Sun, Xiankun Zeng

**Affiliations:** 1 United States Army Medical Research Institute of Infectious Diseases, Fort Detrick, Frederick, MD 21702 USA; 2 Janelia Research Campus, Howard Hughes Medical Institute, Ashburn, VA 20147 USA; 3 Optical Microscopy and Analysis Laboratory, Leidos Biomedical Research, Inc., Frederick National Laboratory for Cancer Research, Frederick, MD 21702 USA; 4 Integrated Research Facility at Fort Detrick, National Institute of Allergy and Infectious Diseases, National Institutes of Health, Frederick, MD 21702 USA

## Abstract

Zika virus (ZIKV) is an emerging flavivirus that caused thousands of human infections in recent years. Compared to other human flaviviruses, ZIKV replication is not well understood. Using fluorescent, transmission electron, and focused ion beam-scanning electron microscopy, we examined ZIKV replication dynamics in Vero 76 cells and in the brains of infected laboratory mice. We observed the progressive development of a perinuclear flaviviral replication factory both in vitro and in vivo. In vitro, we illustrated the ZIKV lifecycle from particle cell entry to egress. ZIKV particles assembled and aggregated in an induced convoluted membrane structure and ZIKV strain-specific membranous vesicles. While most mature virus particles egressed via membrane budding, some particles also likely trafficked through late endosomes and egressed through membrane abscission. Interestingly, we consistently observed a novel sheet-like virus particle array consisting of a single layer of ZIKV particles. Our study further defines ZIKV replication and identifies a novel hallmark of ZIKV infection.

## Introduction

Zika virus (ZIKV) is a mosquito-borne virus infecting humans  that in the past was associated predominantly with rare subclinical infections or self-limiting disease without apparent sequelae in Africa and Asia. Since 2007, ZIKV has been emerging in a dramatic manner, suddenly causing tens of thousands of human infections including severe infections associated with central nervous system (CNS) impairment, such as Guillain-Barré syndrome or fetal microcephaly after congenital transmission^1^.

Flaviviruses typically produce enveloped spherical particles (≈50 nm in diameter) and have linear, nonsegmented, single-stranded, positive-sense RNA genomes containing a single open reading frame of approximately 9–13 kb that encodes (from 5′ to 3′) three structural proteins: capsid (C), premembrane/membrane (prM), envelope (E), and seven non-structural (NS) proteins: NS1, NS2A, NS2B, NS3, NS4A, NS4B, and NS5^2^. The prM and E proteins mediate virion attachment to and fusion with host-cell membranes, whereas the NS proteins have multiple roles, including promoting virus replication and evading the host innate immune response^2^. Upon infection, flaviviruses induce perinuclear membrane rearrangements to create a safe, optimal environment for viral replication, i.e., virus replication factories. Typical changes include appearance of convoluted membranes, formation of invaginated vesicles or clustered double-membrane vesicle packets (Vp), and cellular cytoskeleton alterations. Within Vp, flaviviruses replicate via intermediary synthesis of negative-sense antigenomes. Progeny virions are assembled and released through vesicle pores or protrusions from the endoplasmic reticulum (ER)^3–7^. ZIKV is classified in the family *Flaviviridae*. To date, the mechanisms of the ZIKV lifecycle still remain to be fully elucidated, but ZIKV is known to infect multiple cell types in the human brain, including glial cells, neurons, and neuronal stem cells^8–10^. ZIKV enters cells via receptor-mediated endocytosis^8^. ZIKV genomic RNA is translated into replicative proteins necessary to produce negative-sense antigenomes that serve as templates for the production of progeny genomes^2^. Both steps occur via the production of double-stranded RNA (dsRNA) intermediates. Flavivirus-induced cellular membrane alterations have been classified into two types: invaginated vesicles in connection with convoluted membrane of the ER and double-membrane vesicles^11^.

In this report, we add to previous descriptions of cellular ZIKV replication and ZIKV replication factories^12, 13^. Using antibody and in situ staining techniques and two modalities of electron microscopy, we describe the entire ZIKV lifecycle. We  identify a novel structural feature of ZIKV replication, namely sheet-like virus particle array, in grivet (Vero 76) cells infected with African or Brazilian ZIKV isolates.

## Results

### Zika virus multiplies in perinuclear replication factories in vitro

To study the intracellular dynamics of ZIKV replication, we exposed grivet (Vero 76) cells to the African ZIKV MR 766 strain and harvested the cells at various time points to detect ZIKV genomic RNA and dsRNA intermediates using dual immunofluorescent analysis (IFA) and fluorescence in situ hybridization (FISH) staining and an anti-dsRNA antibody.

**Fig. 1:**
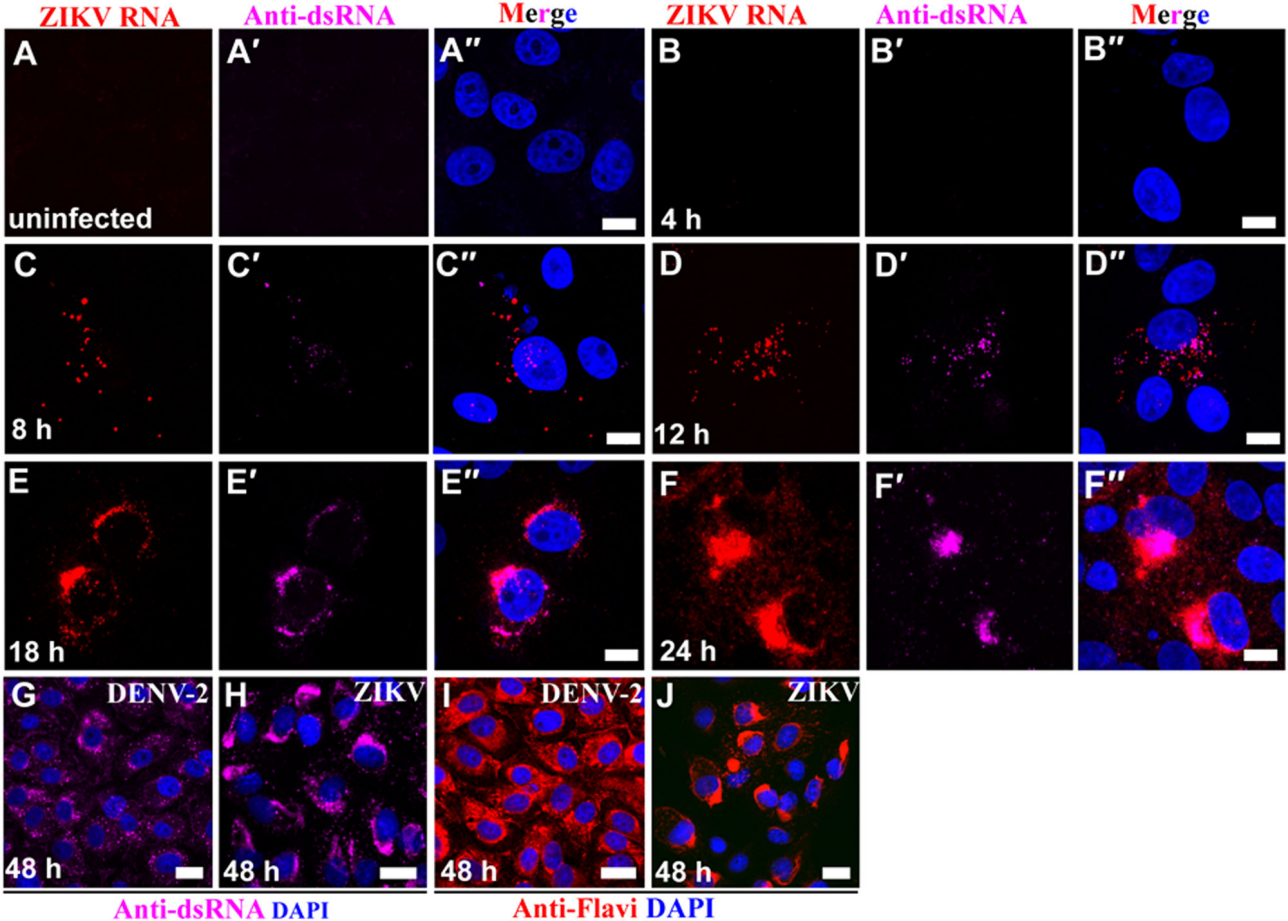
ZIKV multiplies in perinuclear replication factories in vitro. **(a**–**a′′**) Vero 76 control cells (uninfected cells). (**b**–**f′′**) Vero 76 cells were infected with ZIKV African MR 766 strain and IFA/FISH-stained using a probe targeting the ZIKV genome (red) and J2 antibody against dsRNA (magenta) at the indicated time points. (**g**–**j)** Location of ZIKV perinuclear replication factories was  compared with even distribution of DENV-2 in the cytoplasm by staining of antibody J2 against dsRNA (magenta) and antibody against flaviviruses (red). Nuclei were stained with DAPI (blue). Scale bar, 10 µm (**a**–**f**′′) and 20 µm (**g**–**j**)

ZIKV genomic RNA and dsRNA were not detectable in the cytoplasm of Vero 76 cells until 8 hours (h) post-exposure (PE) (Fig. 1a–c′′). Both genomic RNA and dsRNA were scattered throughout the cytoplasm. However, beginning at 12 h PE, ZIKV multiplication was robust in the perinuclear cytoplasm and resulted in the formation of large ZIKV inclusion/replication factories. At these time points, ZIKV replication was restricted to the perinuclear region and to the center regions of viral inclusion bodies (Fig. 1d–f′′). Neighboring cells also became infected by 18 h PE (Supplementary Figure S1A–1E). By 48 h PE, ZIKV infection was evident in nearly every cell (Supplementary Figure S1D). By 72 h PE, cell growth stalled (Supplementary Figure S1E), and the number of viable cells rapidly decreased as ZIKV-induced cytopathic effects (e.g., sloughing of cells, nuclear fragmentation) developed. These effects were most likely due to ZIKV-induced apoptosis as suggested by elevated expression of cleaved caspase 3 (Supplementary Figure S1F).

Interestingly, in comparison to ZIKV genomic RNA, ZIKV antigen could not detected by IFA with anti-flavivirus antibody until 18 h PE (Supplementary Figure S2). Once detected, ZIKV antigen was mainly detected in the perinuclear cytoplasm and in replication factories similar to ZIKV genomic RNA (Supplementary Figure S2). However, in  comparison to ZIKV distribution, dengue virus 2 (DENV-2), another flavivirus closely related to ZIKV, was distributed evenly in the whole cytoplasm even at 48 h PE (Fig. 1g–j). Together, these data indicate that ZIKV replication factories gradually emerge in the perinuclear cytoplasm of ZIKV-infected Vero 76 cells.

### ZIKV multiplies in replication factories of diverse brain cell types in infected mice

To determine if replication factories are also a hallmark of ZIKV infection in vivo, we investigated virus replication in the brains of ZIKV-infected C57BL/6 laboratory mice treated with intraperitoneal  (IP) injection of Mab-5A3 antibody (Leinco Technology, St. Louis, MO) to disrupt type I interferon  signaling^14^.

**Fig. 2:**
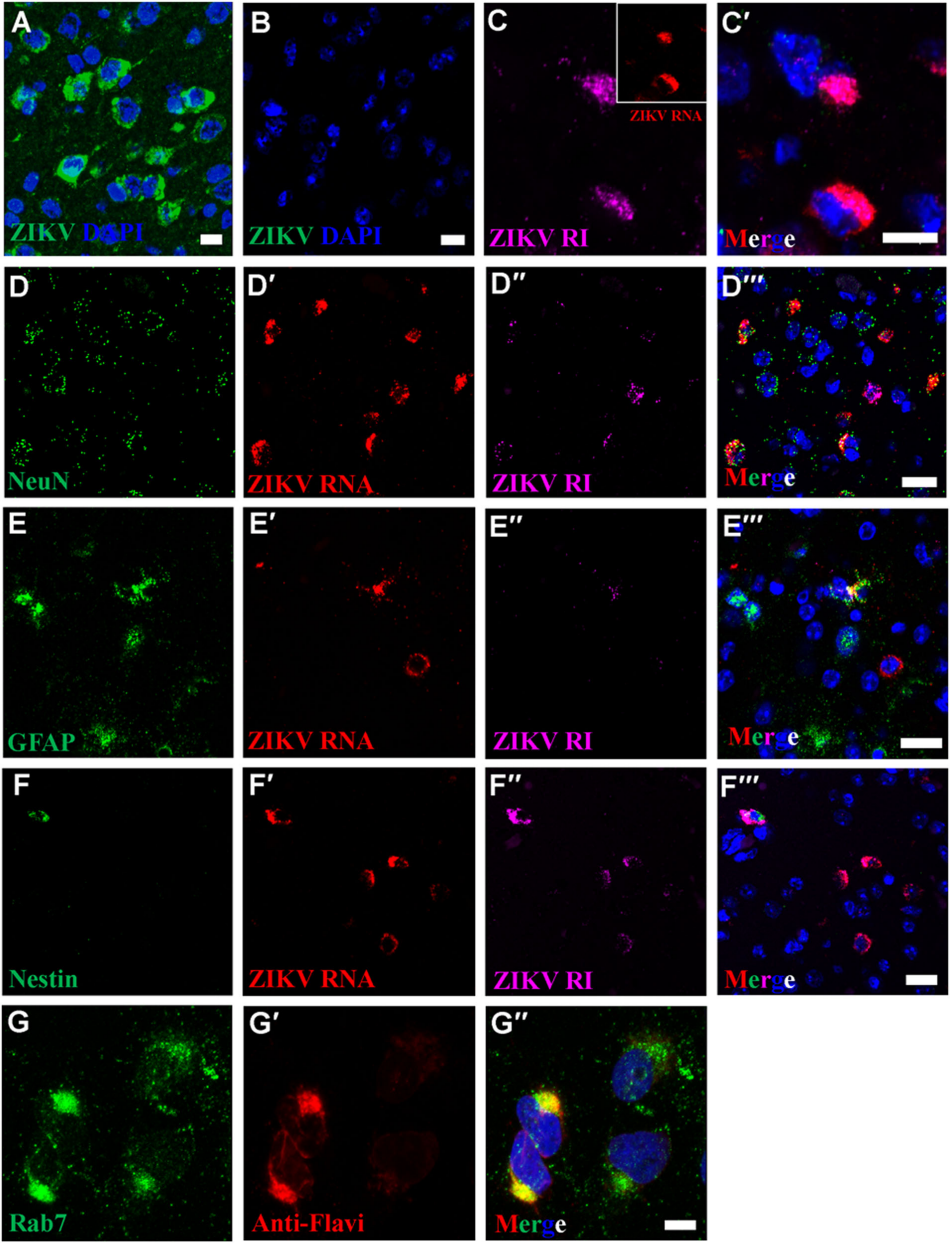
ZIKV multiplies in replication factories of diverse brain cell types in C57BL/6 laboratory mice 7 or 8 days after IP  exposure to 6.4 log_10_ PFUs of ZIKV strain DarArD 41525. (**a**–**b**) IFA using anti-ZIKV E antibody reveals perinuclear ZIKV replication factories in infected (**a**), but not in uninfected mouse brains (**b**). **c**–**c′** Perinuclear ZIKV replication factories were  revealed by multiplex FISH detecting ZIKV genomic (inset, red) or antigenomic RNA (magenta). (**d**–**f′′)** Multiplex FISH detection of ZIKV replication factories in neurons (*Rbfox3/NeuN*), glial cells (*GFAP*), and neuroectodermal stem cells (*Nes*). (**g**–**g′′)** Colocalization of ZIKV particles and endosomes were revealed by IFA using anti-flavivirus antibody (red) and anti-Rab7 antibody (green). Nuclei were stained with DAPI (blue). Scale bar, 10 µm (**a**–**c′**, **g**–**g′**) and 20 µm (**d**–**f′**)

In this mouse model^14^, we previously observed significant pathology and numerous ZIKV particles in brains of mice that succumbed of disease. Using an antibody against the ZIKV E protein in the IFA, we then detected perinuclear ZIKV antigen in many different brain cell types of mice infected with ZIKV but not in mock-infected mice (Fig. 2a, b). Multiplex FISH targeted ZIKV genomic and antigenome RNA (Fig. 2c–c′), in conjunction with probes against cell type-specific markers: glial fibrillary acidic protein (GFAP, glial cells), RNA-binding protein, Fox-1 homolog 3 (Rbfox3) (neurons), and Nes (neuronal stem cells). Consistent with our in vitro findings, we detected active ZIKV replication in perinuclear viral factories of all three cell types (Fig. 2d–f′′′). Interestingly, we further observed that the ZIKV perinuclear viral factories were highly co-localized with late endosomes (identified using anti-Rab7 antibodies, Fig. 2g–g′′). Together, these data support the notion that perinuclear viral factories serve to facilitate the efficient replication and assembly of ZIKV both in vitro and in vivo.

### ZIKV induces intracellular membrane reorganization

**Fig. 3:**
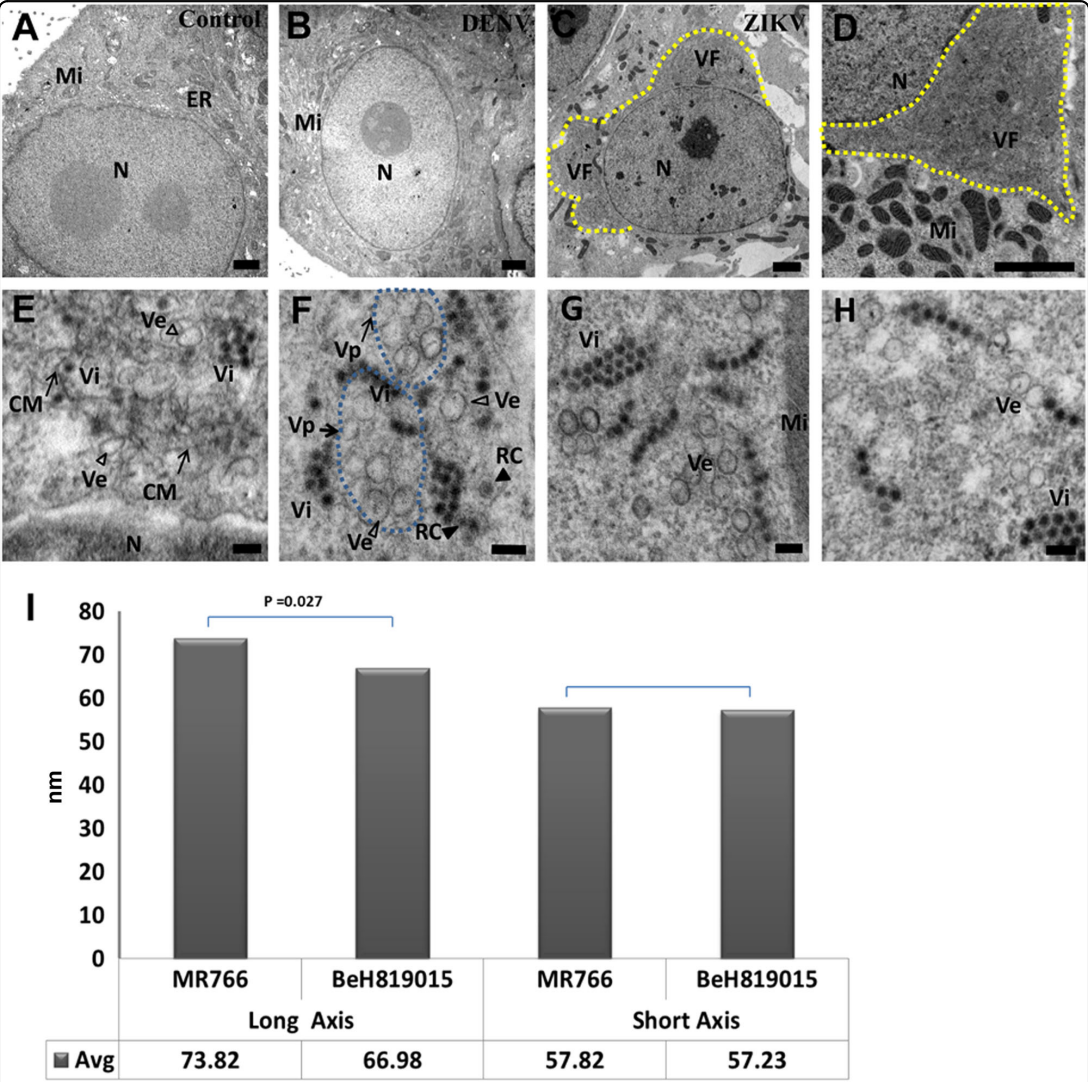
ZIKV induces intracellular membrane reorganization. ZIKV-infected cells were analyzed 48 h post-exposure and analyzed by TEM. (**a)** In the perinuclear region of uninfected Vero 76 cell, endoplasmic reticulum (ER) membranes are organized in orderly fashion. Mi, mitochondria; N, nuclei. **(b)** In DENV-2-infected Vero 76 cells, convoluted ER membranes are evident, but visible perinuclear replication factories are lacking. **(c**) Perinuclear ZIKV replication factory (VF, yellow dashed line) in the degenerated rough ER. (**d)** Multiple mitochondria (Mi) in proximity to the ZIKV replication factory. Large numbers of ZIKV particles are visible in both panels (tiny, dark pin dots). (**e**, **f)** Convoluted membranes (CM, black arrows) and vesicular structures (empty arrowheads) are located within ZIKV replication factories. Aggregated vesicles (Ve) were wrapped in a packet (Vp, black arrows, blue dashed line). Note the likely presence of replication centers (RC, solid arrow heads) in two of the vesicles and newly assembled clusters of virions (Vi). Scale bar, 2 µm (**a**–**d**), 500 nm (**e**), and 100 nm (**f**–**h**). (**g**, **h)** Representative images of induced vesicles inside cells infected with ZIKV MR766 (**e**) and BeH819015 strains (**f**). **(i)** Measurement of vesicle sizes induced by ZIKV MR766 (*n* = 162) and BeH819015 strains (*n* = 44)

To further understand the characteristics of ZIKV-induced membrane changes, ZIKV-infected Vero 76 cells were fixed at 48 h PE and resin-embedded for transmission electron microscopy (TEM) examination. In agreement with IFA/FISH findings, virus factories were observed along the edge of host-cell nuclei, usurping a large portion of the distended rough ER in 50 of 53 (94%) of  ZIKV-infected cells (Fig. 3c) but not in uninfected control (Fig. 3a) using TEM. In contrast, obvious virus factories were not observed in all 52 examined DENV-2-infected cells (Fig. 3b). Numerous mitochondria aggregated in close proximity to viral replication factories (Fig. 3d). The viral replication factories were composed of convoluted membranes that contain multiple vesicular structures (Fig. 3e). Higher magnification revealed a complex set of special membranous structures as a  consequence of ZIKV-induced membrane alterations. The single membrane vesicles and, probably, more advanced replication complexes (replication centers) in some of the vesicles were enclosed inside membrane-bound Vp. Virus particles were located in clusters or paracrystalline arrays neighboring Vp, likely released after finishing assembly inside the packet (Fig. 3f). In the case of other flaviviruses, these structures are the sites for genomic RNA synthesis and virus particle assembly^15^.

Interestingly, it was recently suggested that the size of ZIKV-induced vesicles not only varies by host cell type but also by ZIKV strain^12^. We confirm these findings for Vero 76 cells infected with either an African or a Brazilian ZIKV strain (Fig. 3g–i). The Brazilian BeH819015 strain-induced vesicles were largely spherical with an average long-axis-to-short-axis ratio of 67:57 (average diameter: 66.98 ± 2.90 and 57.23 ± 2.83 nm, *n* = 44). The average ratio of African MR 766 strain-induced vesicles, however, was 74:58 (average diameter: 73.82 ± 1.38 and 57.82 ± 1.13 nm, *n* = 162), indicating that MR 766-induced vesicles are ovoid. Together, our data support the notion that ZIKV induces subcellular changes similar to those described for other flaviviruses.

### The ZIKV lifecycle

**Fig. 4:**
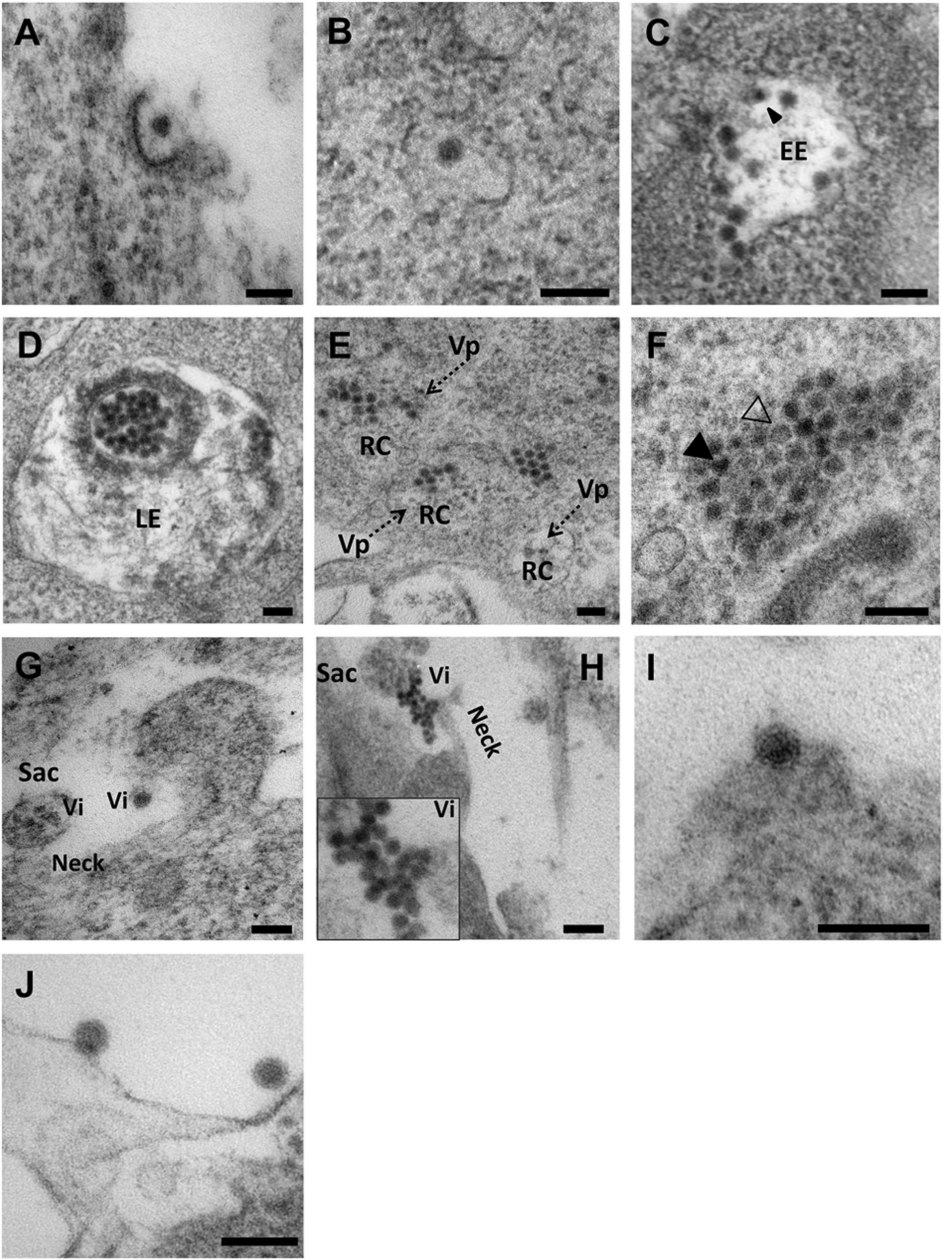
Uptake, replication, and egress of ZIKV from inside the cells. **(a)** ZIKV particle cell entry. Note the thickening of the membrane and formation of a clathrin-coated pit. **(b)** A ZIKV particle enters the host cell via formation of a clathrin-coated vesicle. (**c**) The ZIKV particles (arrow heads) are leaving early endosomal (EE) vacuoles. (**d**) A late endosome (multivesicular body [MVB]) with assembled virus particles. (**e**) Replication centers (RC) inside vesicles wrapped in packets (Vp, black dashed arrows). **(f**) ZIKV paracrystalline array in the ER lumen. Note incompletely assembled particles (partial or empty: empty arrowhead) next to mature particles (solid arrowhead). (**g**) An MVB sac containing vesicles about to be abscised from the neck of the cytoplasmic membrane. **(h)** Virions (Vi) egressing from the MVB sac. Inset shows virons at high magnification. **(I)** Mature virus particle budding from the cytoplasmic membrane. **(j)** Released virus particles in the extracellular space. Scale bar, 100 nm

In our studies, the initial contact between ZIKV particles and the host-cell membrane led to the thickening at sites of membrane invaginations and the appearance of clathrin-coated pits (Fig. 4a). ZIKV particles then appeared to fuse with the endosomal membrane to form endocytic vesicles (Fig. 4b). Multiple virus particles entered the early endosomes via vesicle fusion (Fig. 4c). A late endosome-like structure (or multivesicular body) was observed containing two groups of newly assembled virus particles (Fig. 4d) that transported ZIKV RNA to the ER. Mature virus particles were then assembled in ER-derived organelle-like structures or virus factories through membrane invaginations. These particles either budded into the ER lumen in a fashion similar to that observed with DENVs  or through the formation of double-membrane vesicles and budding into the cytosol similar to hepatitis C virus (HCV)^11, 16^. Virus replication likely took place at the replication centers inside the vesicles enclosed by Vp. Virus particles of various degrees of maturation accumulated, assembled, and were released from Vp (Fig. 4e). Newly synthesized virus particles aggregated and formed paracrystalline arrays in the distended lumen of the ER. Some of the electron-transparent particles appeared to be incompletely assembled (Fig. 4f).

Interestingly, a large number of newly assembled ZIKV particles were cloaked inside late endosome-like vacuoles, indicating they were ready to be transported via a multivesicular body secretory pathway for viral egress (Fig. 4d). Upon finishing assembly in the ER, many progeny virus particles accumulated in a multivesicular body-like structure. The multivesicular body seemed connected to the ER through a neck-like channel that exited into the cytoplasm. Virions likely exited through abscission from the membrane or through a pore on the multivesicular body or sac (Fig. 4g, h). This observation, combined with our data supporting co-localization of ZIKV antigen with late endosomes indicate the involvement of endosomes in the ZIKV replication cycle. ZIKV particle budding was also observed in other areas of the ER and away from Vp (Fig. 4i), indicating that mature ZIKV virions trafficked through a conventional secretory pathway for release from the cell (Fig. 4j).

### A sheet-like virus particle array is a hallmark of ZIKV infection

**Fig. 5:**
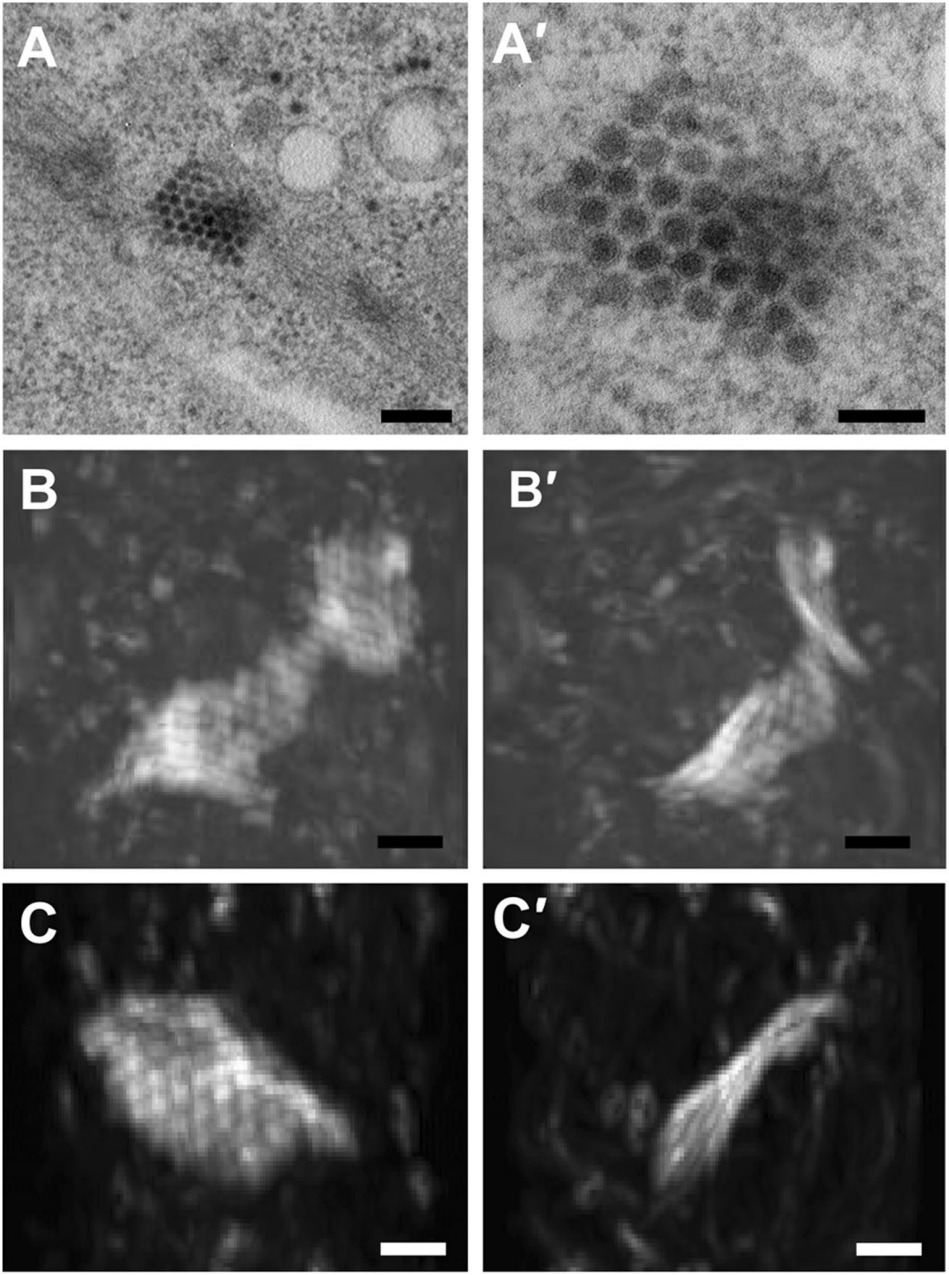
Sheet-like ZIKV particle array. **(a**–**a′)** ZIKV paracrystalline (PC) array structure at low and high magnifications of  transmission electron micrographs (TEM). **(b**–**b′)** Reconstructed 3D images from focused ion beam scanning electron microscopy (FIB-SEM) at two different angels reveal a wavy sheet-like array of a single layer of ZIKV particles. **(c**–**c′**) Another sheet-like ZIKV particle array structure at two different angles. The clockwise rotation angles of **(b′)** and (**c′**) relative to **(b)** and (**c**) are 110 degrees around the vertical axis. Scale bar, 200 nm (**a**) and 100 nm (**a′**–**c′**)

Using electron tomography based on 250-nm sections,researchers  recently suggested that ZIKV particles aggregate inside the ER in the form of a grape-like clump^12^. However, detailed 3D information for the entire infected cell has not been obtained. Focused ion beam scanning electron microscopy (FIB-SEM) is a technique that enables the reconstruction of 3D electron-microscopic images to achieve cellular and tissue level volume observation^17–19^. Consequently, we used FIB-SEM to visualize the observed ZIKV-induced paracrystalline arrays observed during our in vitro studies. The paracrystalline arrays were located mostly in the proximity of putative vesicles that harbor ZIKV replication centers (Fig. 5a). The arrays contained some fully assembled, enveloped virus particles with electron-dense viral genomes, but also enveloped particles clearly lacking RNA (empty virus particles) (Fig. 5a′). The reconstructed 3D volume of the paracrystalline array revealed a wavy sheet-like structure composed of a single layer of virus particles (Fig. 5b–c′ and Supplementary Movies 1 and 2). This finding indicates that the ZIKV particles aggregated in a form of lattice planes rather than grape-like clumps after release into the ER lumen.

## Discussion

Numerous ultrastructural studies have shown previously that positive-strand RNA viruses are synthesized and assembled in the cytoplasmic area surrounding the nucleus at the ER and Golgi apparatus. The assembly involves a process called cellular membrane rearrangement that leads to the biogenesis of specific intracellular virus replication and particle assembly compartments^3–7^. Rearrangement of cytoplasmic membranes and the participation of many cellular components are attributed to form specific compartments that facilitate virus replication. Flaviviruses induce at least three membrane changes: convoluted membranes, paracrystalline vesicle arrays, and Vp. Paracrystalline vesicle arrays were connected to membrane rearrangements in cells infected by Kunjin virus and tick-borne encephalitis virus. These arrays are thought to be sites of the viral polyprotein processing based on the absence of viral RNA^20–22^. On the other hand, paracrystalline arrays consisting of partially or fully assembled flavivirus particles have long been known to be induced by DENVs ^23^, Japanese encephalitis virus^24^, and St. Louis encephalitis virus^25^.

Relatively little is known about the replication of ZIKV and how it compares to other flaviviruses. We used fluorescence (IFA and FISH) and electron microscopy (TEM and FIB-SEM) to validate previous findings that ZIKV replication in perinuclear replication factories in various mouse brain cell types in vivo and in infected cells of different origins in vitro^14, 26–29^. We were unable to find paracrystalline vesicle arrays, but paracrystalline ZIKV arrays containing mature and empty particles were abundant in the ER near the viral replication centers. We characterized for the first time the paracrystalline ZIKV particle arrays in three dimensions at high resolution, and discovered this array to be a novel sheet-like structure composed of a single layer of ZIKV particles rather than a grape-like crump, as suggested previously^12^. Results of future studies will determine whether this sheet is a unique feature of ZIKV infections or ZIKV infections in particular cell types, and whether this sheet has any biological relevance or is merely a byproduct of virus replication. Our results demonstrate that ZIKV perinuclear replication factories develop rapidly after the initial phase of infection. A large number of mitochondria aggregated in close proximity to viral replication factories (Fig. 3d), implying an increased energy need for virus replication. ZIKV replication and particle assembly appear to follow a path that is sophisticated and more like that of the closely related DENVs (flaviviral genus *Flavivirus*) than that of the more distantly related HCV (flavivirus genus *Hepacivirus*). DENVs  induce similar membrane rearrangements and invagination of ER, and, thus, such viruses create vesicles containing viral dsRNA and replication proteins that are also enclosed with membrane packets. In contrast, HCV induces formation of double-membrane vesicles in which virus RNA replicates, eventually assembles, and releases by protruding through the ER^11^.

In contrast to a previous report^12^, results of our studies indicate that different ZIKV strains may cause distinct subcellular rearrangements in vitro. Our data intriguingly suggest that newly assembled ZIKV particles could possibly use the endosomal machinery for egress via abscission from ER membrane necks, escaping from the trafficking pathway to lysosomes through an unknown mechanism.

Together, results of our study indicate that although flaviviruses follow a common theme of replication, individual viruses probably cause distinct subcellular rearrangements dependent on the infected animal, the infected cell type, and possibly even dependent on virus strain. Future studies will focus on the pathophysiological consequences, if any, of these differences.

## Materials and methods

### Cells and viruses

Grivet (*Chlorocebus aethiops*) Vero 76 kidney cells (ATCC, Manassas, VA; #CRL-1587) were maintained in Eagle’s minimum essential media (EMEM; Mediatech, Manassas, VA) supplemented with 10% heat-inactivated fetal bovine serum Hyclone, Logan, UT), 1% l-glutamine (Hyclone), 1% non-essential amino acid solution (MilliporeSigma, Temecula, CA), 1% Hepes (MilliporeSigma), and 1% penicillin-streptomycin solution (Mediatech) at 37 °C in a 5% CO_2_ atmosphere. African Zika virus (ZIKV) strain MR 766 (1947) was obtained from ATCC (#VR-84; GenBank #AY632535; RefSeq #NC_012532.1). Brazilian ZIKV strain BeH819015 (2015; GenBank #KU365778.1) was generated by reverse genetics. The complete genome was commercially synthesized and maintained as three sub-genomic plasmids. To rescue virus, the subgenomic fragments were amplified by polymerase chain reaction using high-fidelity Phusion polymerase (NEB, Ipswich, MA) and joined into a full genome construct by Gibson assembly (NEB). The Gibson assembly reaction was used as a template in a subsequent polymerase chain reaction to reamplify the full genome expression construct to obtain sufficient template for in vitro transcription. Complete genome RNA was generated by *in vitro* transcription (NEB) and transfected into Vero 76 cells to generate virus. DENV-2 was obtained from ATCC (#VR-1584). Viruses were added to cell cultures at a multiplicity of infection (MOI) of 0.2. Cells were then incubated for 1 h for virus adsorption, washed with medium, and maintained in EMEM with 2% fetal bovine serum. The infected cells were fixed at the indicated h post-exposure.

### Dual in vitro staining

Vero 76 cells were exposed to ZIKV strains at an MOI = 0.2 for 1 h at 37 °C. Cells were washed with EMEM and fixed in 10% formalin for 30 minutes  (min), rinsed with RNase-free phosphate-buffered saline (PBS), digested using 5 µg/ml of proteinase K in TE buffer (50 mM of Tris base, 1 mM of ethylenediaminetetraacetic acid, 0.5% Triton X-100, pH 8.0) at 37 °C for 15 min. Cells were rinsed in PBS and then PBTH (PBS containing 0.1% Tween-20, 50 µg/ml of heparin, and 250 µg/ml of tRNA) and incubated with primary antibodies: mouse anti-dsRNA J2 (English and Scientific Consulting Kft., Szirak, HU) 1:500; mouse anti-flavivirus group antigen antibody, clone D1-4G2-4-15 (MilliporeSigma) 1:500; rabbit anti-Rab7 (Cell Signaling Technology, Danvers, MA) 1:200; and rabbit anti-caspase 3 (Cell Signaling Technology) 1:200. Cells were then diluted in PBTHR (PBTH containing 0.2 U/ml of RNase inhibitor and 1 mM of dithiothreitol) at 4 °C overnight. After a brief wash in PBTH, cells were incubated with fluorescent secondary antibody (goat anti-mouse Alex Fluor 488 or goat anti-rabbit Alex Fluor 488, Life Technologies, Carlsbad, CA) in PBTHR for 1 h at room temperature. Cells were fixed in 10% formalin/PBS for 20 min to crosslink primary and secondary antibodies to their cognate antigens for IFA. FISH was performed using the RNAscope® Fluorescent Multiplex Kit (Advanced Cell Diagnostics, Newark, CA) according to the manufacturer’s instructions with minor modifications. Twenty ZZ probe pairs with C1 channel (red) targeting the ZIKV genome were synthesized by Advanced Cell Diagnostics (Cat# 463781). Cells stained by IFA were incubated with ZIKV probe pairs at 40 °C in a hybridization oven for 2 h. After rinsing with PBS, FISH signal was amplified using company-provided Pre-amplifier and Amplifier conjugated to red fluorescent dye. Cells were counterstained with 4′,6-diamidino-2-phenylindole (DAPI), mounted, and stored at 4 °C until image analysis. IF and FISH images were captured on an LSM 780 Confocal Microscope (Zeiss, Oberkochen, Germany) and processed using open-source ImageJ software (National Institutes of Health, Bethesda, MD; https://imagej.nih.gov/ij/).

### Animal experiments

The animal experiment was previously described^14^. Briefly, 10 female C57BL/6 laboratory mice per group, 5 weeks of age, were treated with 3 mg of monoclonal antibody 5A3 to block type I IFN signaling or with PBS (total volume: 200 µl) on day 1 prior to IP exposure to 6.4 log_10_ plaque-forming units (PFUs) of ZIKV strain DarArD 41525 on day 0. Treatment with monoclonal antibody 5A3 or saline continued on days +1 or +4 PE. Mice were euthanized at day 7 or 8 PE. The brain tissues were fixed in formalin and embedded in paraffin for sectioning. All animal studies, including animal husbandry, virus inoculation, animal euthanasia, sample collection, creation of formalin-fixed paraffin-embedded (FFPE) tissues, and sectioning were completed at USAMRIID.

The animal studies were conducted under an Instituional Animal Care and Use Committee- approved protocol in compliance with the Animal Welfare Act, Public Health Service Policy, and other Federal statutes and regulations relating to animals and experiments involving animals. The facility where this research was conducted is accredited by the Association for Assessment and Accreditation of Laboratory Animal Care, International and adheres to principles stated in the Guide for the Care and Use of Laboratory Animals, National Research Council, 2011.

### In vivo staining

Multiplex FISH was performed using the RNAscope® Fluorescent Multiplex Kit (Advanced Cell Diagnostics) according to the manufacturer’s instructions with minor modifications. Twenty ZZ probe pairs with C1 channel (red) targeting the ZIKV genome (Cat# 463781), forty ZZ probe pairs with C2 Channel (green) targeting the mouse *Rbfox3*/*NeuN* (Cat# 481701), forty ZZ probe pairs with C2 Channel (green) targeting the mouse *GFAP* (Cat# 481691), forty ZZ probe pairs with C2 Channel (green) targeting the mouse nestin gene *Nes* (Cat# 481741), and forty ZZ probe pairs with C3 channel (magenta) targeting the ZIKV replicative intermediate (RI; Cat# 467918) were synthesized by Advanced Cell Diagnostics. Rabbit anti-Zika virus envelope glycoprotein antibody was purchased from IBT Bioservices (Gaithersburg, MD); FFPE laboratory mouse brain tissue sections were deparaffinized with xylene and a series of ethanol washes as previously described. Tissue sections were treated with 0.1% Sudan Black B (Sigma-Aldrich, St. Louis, MO) to reduce autofluorescence, heated by boiling in Antigen Retrieval Buffer, and digested with proteinase K (Advanced Cell Diagnostics). Sections were then processed using the procedures outlined above.

### Transmission electron microscopy

Vero 76 cells grown on the coverslips of MatTek glass bottom dishes (MatTek Corporation, MA) were exposed to ZIKV and incubated for 48 h at 37 °C and 5% CO_2_. Cells were primary-fixed with 2.5% formaldehyde, 2.5% glutaraldehyde, and 0.1 M of sodium cacodylate pH 7.4 buffer and incubated for 1 h on ice. After washing three times in ice-cold sodium cacodylate buffer (0.1 mM) for 3 min each, the primary-fixed cells were post-fixed by incubation with 1% osmium tetroxide in 0.1 M of sodium cacodylate for 1 h on ice. After washing with distilled water three times for 3 min each, fixed cells were stained and stabilized in ice-cold 1% uranyl acetate for 1 h and dehydrated in an ice-cold series of 25, 50, 75, and 95% ethanol successively for 3 min each. Cells were then dehydrated at room temperature three times for 3 min each in 100% ethanol and infiltrated with well-mixed 50% ethanol, 50% Durcupan ACM resin (Sigma-Aldrich) for 1 h with agitation at room temperature followed by 100% Durcupan ACM resin twice for 3 h with agitation. Infiltrated samples on glass coverslips of the MatTek dishes were placed in a hybridization oven for polymerization at 60 °C for at least 48 h. Glass coverslips were peeled away from the bottom of the MatTek dishes using razor blades and were cut out randomly into small pieces. The pieces were then glued with the cell side upwards to a dummy block for sectioning. Thin sections (≈80 nm thick) were collected and pre-stained with 1% uranyl acetate and Sato lead before examination in a JEOL 1011 transmission electron microscope (JEOL, Peabody, MA) at 80 kV. Digital images were acquired using the Advanced Microscopy Techniques camera system (Advanced Microscopy Techniques, Woburn, MA).

### Focused ion beam scanning electron microscopy

Sample block preparation was identical to the method described above for transmission electron microscopy. Heavy metal-stained and plastic-embedded samples were trimmed to 50 × 40 × 6 µm^3^ areas with isotropic 8-nm pixels that encompassed several cells. Samples were visualized by scanning electron microscopy using the Zeiss NVision 40 system (Zeiss, Thornwood, NY) operating at 1.1 kV. A few sub-volumes were cropped to illustrate the ZIKV particle organization in 3D movies using the open source software Fiji 11 inspired by the US National Institutes of Health (https://imagej.net/Fiji/Downloads).

## Electronic supplementary material

Supplementary Figure S1

Supplementary Figure S2

Supplementary figure legends

Supplementary Movie 1

Supplementary Movie 2
