# Supplementary figures and images for: A novel sheet-like virus particle array is a hallmark of Zika virus infection
Source: Emerg Microbes Infect. 2018 Apr 25;7:69. doi: 10.1038/s41426-018-0071-8 (PMC5915449; doi:10.1038/s41426-018-0071-8)

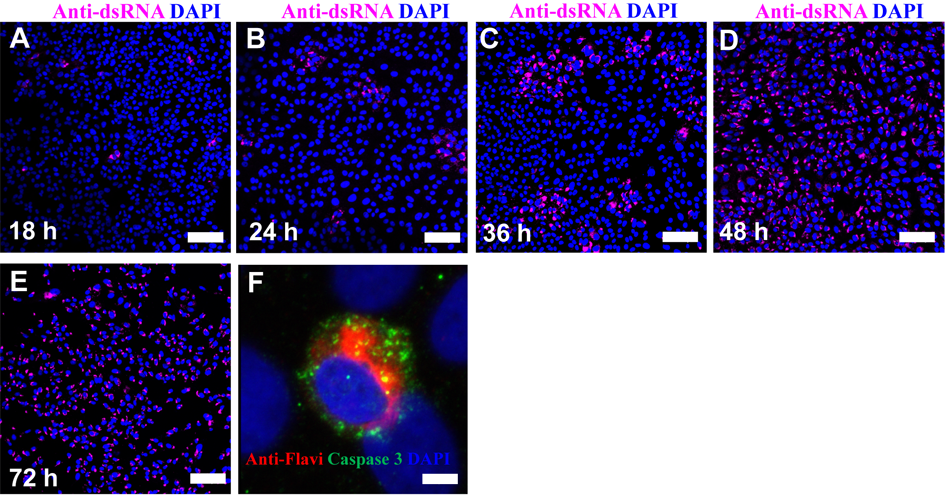

Supplement: Supplementary file 1 — Supplementary Figure S1 [file 41426_2018_71_MOESM1_ESM.tif]

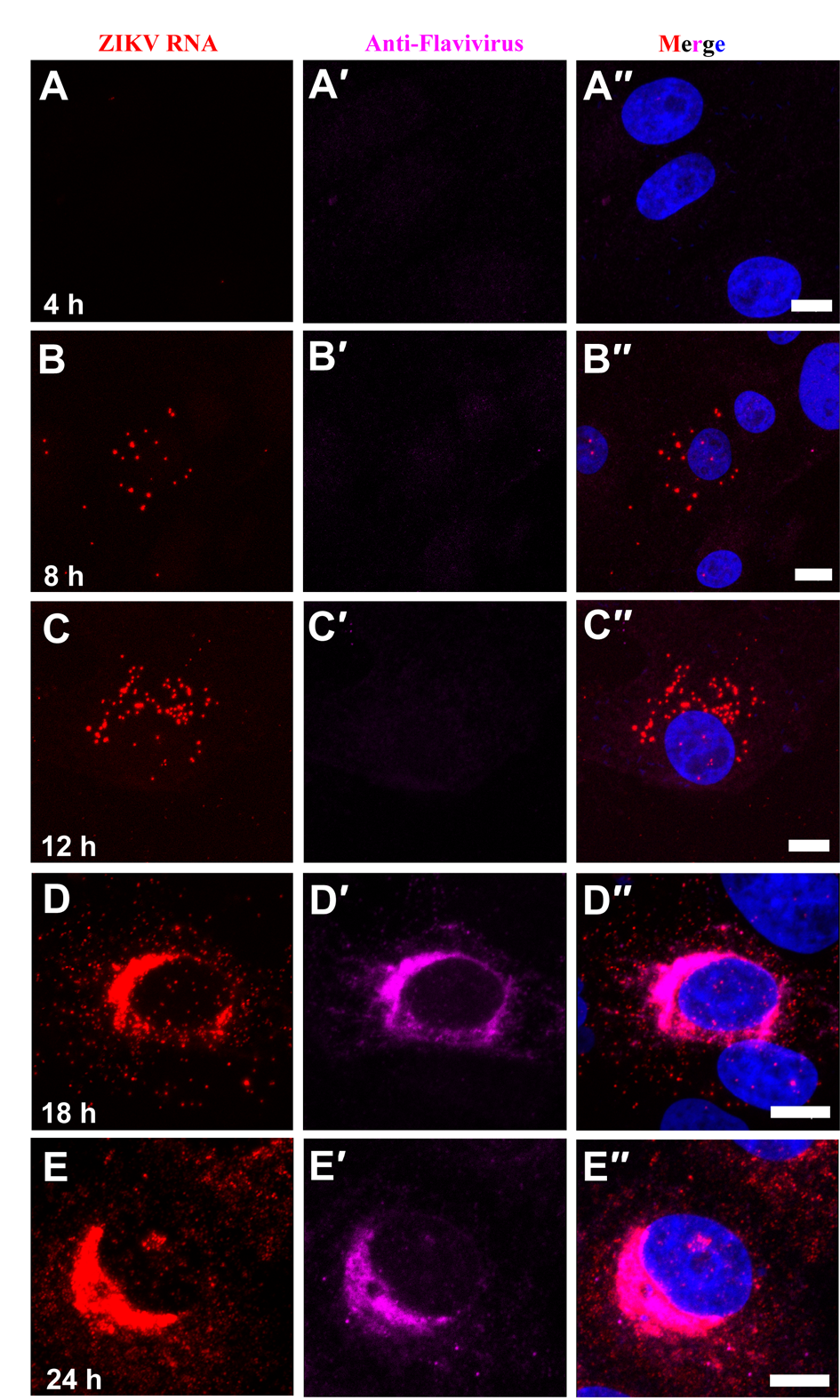

Supplement: Supplementary file 2 — Supplementary Figure S2 [file 41426_2018_71_MOESM2_ESM.tif]
