# Supplementary material for: A novel sheet-like virus particle array is a hallmark of Zika virus infection
Source: Emerg Microbes Infect. 2018 Apr 25;7:69. doi: 10.1038/s41426-018-0071-8 (PMC5915449; doi:10.1038/s41426-018-0071-8)
Supplement: Supplementary file 3 — Supplementary figure legends [file 41426_2018_71_MOESM3_ESM.docx]

**Supplementary Figure legends**

**Figure S1** **Kinetics of the African ZIKV strain MR 766 replication.** (**A–E**) Vero 76 cells were infected with ZIKV at an MOI of 0.2, fixed, and stained by J2 anti-dsRNA (magenta) at indicated times. (**F**) Detection of cleaved Caspas-3 (green) in ZIKV (anti-flavivirus, red) infected Vero cells indicating apoptotic cell death. Nuclei were stained with DAPI (blue). Scale bar, 100 µm in A–E and 5µm in F.

**Figure S2** **Detection of ZIKV RNA and protein in Vero 76 cells**. Vero 76 cells were infected with African ZIKV strain MR 766 at an MOI of 0.2 and fixed at indicated times. Dual FISH and IFA assay was performed using probe targeting ZIKV genomic RNA (red) and anti-flavivirus antibody (magenta). Nuclei were stained with DAPI (blue). Scale bar, 10 µm in A–E′′.
